# Research on Pricing and Service Level Strategies of Dual Channel Reverse Supply Chain Considering Consumer Preference in Multi-Regional Situations

**DOI:** 10.3390/ijerph17239143

**Published:** 2020-12-07

**Authors:** Yao Kang, Juhong Chen, Di Wu

**Affiliations:** School of Economics and Management, Xi’an University of Technology, Xi’an 710048, China; kangyao6077@163.com (Y.K.); wdfrancis@163.com (D.W.)

**Keywords:** pricing strategies, service level, dual channel reverse supply chain, Stackelberg game, multi-regional situation

## Abstract

Facing the increasingly serious waste electrical and electronic equipment (WEEE) recycling problem, recycling enterprises actively introduce online recycling channels, build dual channel reverse supply chains (DRSC), and use high-level recycling price and service levels to enhance consumers’ recycling enthusiasm and recycling amount. Nevertheless, in China, where the imbalance of regional development is widespread, the recycling center, third-party recycler (TPR), and third-party platform (TPP) are faced with the choices of pricing and service level when facing multi-regional consumers. This paper mainly answers the following questions: (1) When the recycling center and TPP introduce online recycling channels in multi-regional situations, how should they set online recycling price, transfer price, and service level? (2) When consumer preference for online channels changes in a certain region, how should recycling enterprises adjust their optimal pricing and service level decisions for different regions to maximize their own profits? How do the profits of recycling enterprises change? In order to solve the above problems, in this paper, we propose three pricing and service level decision models for the recycling center with online channels, namely, keeping prices unchanged, unifying all prices, and maximizing its own profits. By using the Stackelberg game to solve the model, we get the optimal pricing, service level decisions, as well as the maximum profits of the recycling center, TPP, and TPR when consumer preference changes. By analyzing the results of the model, we find that the change of consumer preference for online channels in a certain region will affect the decision and profits of multi-regional recycling enterprises. Specifically, consumer preference for online channels in a certain region will not only lead to an increase in the profits of the recycling center and TPP and a decrease in the profit of local TPRs, but also an increase in the profit of TPRs in other regions. In addition, at the beginning of introducing online channels, the recycling center can adopt two strategies to avoid conflicts among channels: keeping offline transfer prices unchanged and unifying all transfer prices, but the former promotes its economic profits more significantly.

## 1. Introduction

Due to its great influence in solving the problems of resource shortage and environmental degradation, dual channel reverse supply chain (DRSC) has attracted much attention both in the recycling industry and in academia [1]. Compared with the traditional single-channel reverse supply chain, DRSC, which introduces online recycling channels, attracts a large number of waste electrical and electronic equipment (WEEE) consumers with its high level of recycling price and service [2]. Take Aihuishou, one of the largest recycling centers in China, as an example. In 2017 alone, they recycled more than 5.3 million WEEE, with more than 30,000 active users [3]. In online recycling channels, consumers can communicate with the recycling platform anytime and anywhere, bargain according to the usage time, wear degree, and performance of WEEE, and choose to hand over goods by mail or courier door-to-door, without being constrained by the time and space in offline recycling channels. At the same time, the professional decomposition and disassembly technology of the recycling platform can also avoid the potential risk of infringing consumers’ privacy by informal third-party recyclers (TPRs) in offline channels [4]. Finally, because it is not a multi-level supply chain like that in offline channels, online recycling prices through direct transaction are also significantly higher. Although online channels have many advantages, traditional offline channels still occupy the main proportion of the recycling market, so the recycling center needs to develop dual-channel models based on both offline and online channels. Up to now, academic circles have carried out some research on DRSC’s pricing strategy, contract coordination, and channel competition, so as to continuously improve the profits of recycling enterprises and the recycling amount of WEEE [5].

In the research of pricing decisions for DRSC, enterprise managers and scholars have noticed that regional differences are important factors affecting enterprise decision. As a matter of fact, for a long time the problem of regional differences has always been concerned in both positive and reverse supply chains, especially in a country with extremely unbalanced regional development, such as China [6]. This is because the introduction of online channels not only causes direct competition with offline channels, but also leads to indirect competition among offline channels in different regions. In addition, in terms of pricing decision, although the recycling prices set by the recycling center in online channels are uniform when facing consumers in different regions, the offline recycling prices in different regions are always different under the differentiation strategy [7]. Similar differences in recycling prices in different regions not only appear between different cities in China [8], but even in different areas of the same city, such as Chaoyang District and Haidian District in Beijing. The demand for electronic products, the number of waste products, and consumers’ preferences will all lead to different recycling prices [9]. Therefore, scholars have found that when the factors affecting the pricing decision change in a certain region, in order to maximize their own profits, recycling enterprises not only need to adjust the pricing decision in this region according to the change, but also need to adjust the pricing decision in another region at the same time. Among them, the influence of consumer preference change [10,11,12] for different channels, especially the influence of consumers’ environmental awareness on enterprise decision has been widely studied by scholars [13,14].

On the other hand, contrary to the pricing decision model, the online recycling services provided by the recycling center for consumers in DRSC have obvious regional differences, which are mainly reflected in the responsiveness and convenience of the services [15,16]. Take the service outlets set up by Aihuishou in Beijing and Xi’an, two cities in China, as an example. By 2019, Aihuishou had set up 34 outlets in Beijing and only 10 outlets in Xi’an. The number of outlets directly affects the time taken by couriers to pick up goods after consumers place an order on the app. In offline recycling channels in different cities, because they are very scattered and informal, one might think that their services do not exist or are at a constant and very low level [2]. Although up to now, no scholars have paid attention to the service level decision of enterprises in DRSCs for multi-regional consumers, the level of service will not only affect the recycling amount, but also affect the service cost and enterprise income. Therefore, how to make a comprehensive decision on service level and pricing in DRSC is of great significance. In this context, when consumer preference in a certain region changes, how should the recycling center, third-party platform (TPP), and TPR with different pricing strategies adjust the pricing and service level decision in the region? Do the pricing and service level decisions in another region need to be adjusted? How do their earnings change? These questions are all our focus.

In this paper, we use the Stackelberg game model to study the pricing and service level decision of DRSCs in multi-regional situation. For the recycling center outsourcing the whole online recycling channels to TPPs, we propose three kinds of strategies: keeping offline transfer prices unchanged, unifying all transfer prices, and maximizing its own profit. We use Stackelberg game theory to solve the optimal decision and profit of each recycling enterprise under three kinds of strategies, and verify the results of the above model by using the example analysis to find more research conclusions to provide decision guidance for recycling enterprises. The main contribution of the paper includes the following points. Firstly, it explores the influence of the adjustment of recycling service level on the decision and profit of recycling enterprises and supply chain system in a multi-regional situation in online recycling channels. In the past, no scholars have brought service level decisions into the research of multi-regional supply chain modeling, which is an important discovery in this paper. Secondly, aiming at the realistic situation that there are differences in decisions of recycling enterprises in multi-regional situations, it also studies the influence of consumer preference change on enterprise decision and profit for three types of recycling enterprises. In the past, scholars in related fields only set up models for no more than two types of enterprises considering consumer preference in multi-regional situation. Finally, it compares the differences of decisions and profit trends of enterprises under three pricing decisions in the context of DRSC: keeping the offline transfer price unchanged, unifying all transfer prices, and maximizing their own profits. In fact, although these three pricing models have been widely used in the forward supply chain, they are rarely concerned by supply chain research in the recycling field. The above results can provide theoretical support and decision guidance for recycling enterprises in DRSC.

The reminder of this paper is organized as follows. Section 2 reviews and summarizes relevant literature. Section 3 provides description and parameter design for the model to be established in this paper. In Section 4, we build models and solve them. In Section 5, we verify the results of the model by numerical examples and propose targeted suggestions for enterprise decisions based on the analysis of the results. Finally, in Section 6, we summarize the full text and propose the future research direction.

## 2. Literature Review

In this section, we mainly summarize and organize the past research on DRSC and service level decisions. Within that research, in the process of sorting out the relevant literature on DRSC, we mainly focus on its concept, model, factors affecting decision, etc. In addition, we also analyze the relevant literature on service level decisions from aspects of the concept, classification, and significance to enterprises. 

### 2.1. DRSC

As a relatively new supply chain model, the emergence of DRSC is synchronized with the introduction of online recycling channels based on the Internet. The earliest report on online recycling can be traced back to 2015, when the Chinese government’s Development and Reform Commission advocated exploring innovative models and paths based on “Internet+ recycling” in the 2015 Circular Economy Promotion Plan [17]. However, it was not until 2017 that Feng systematically explored the supply chain model based on online and offline recycling channels for the first time in his paper, and studied the pricing decisions and coordination strategies of the recycling center and TPR by constructing the profit function based on consumer preference [1]. On this basis, Giri (2017) pointed out that the emergence of online recycling channels can significantly improve the recycling conversion rate of waste products, and from the perspective of enterprise dominance, studied the pricing decisions and coordination strategies when different enterprises took the lead in the dual channel closed-loop supply chain with online recycling channels [5]. In 2018, Chen and Wu proposed the term dual channel reverse supply chain and defined it as a reverse supply chain model with both online and offline recycling channels. In addition, they also proposed three types of recycling pricing models according to the differences of consumer preference in different regions [6]. At the same time, in addition to using the construction model to study the decision issues of DRSC, some scholars also use case analysis and action research to explore it. Tong (2018) applied action research to study the online recycling mode based on the Internet, which is divided into online transaction between consumers and recyclers, intelligent recycling machine recycling, and community recycling based on garbage classification, and pointed out that these online recycling modes can significantly improve the convenience for consumers [18]. Wang (2018) surveyed ten representative online recycling enterprises in China, and summarized four representative online recycling modes, namely chain operation mode, O2O (Online to Offline) platform transaction mode, community mode, and intelligent recycling machine mode, and put forward specific suggestions for their future development [19]. Qu (2019) pointed out through research that recycling services are an important advantage of online channels, and through consumer survey that recycling price, recycler, and recycling amount are the biggest concern for consumers in online recycling channels [20]. 

Although all the above literature uses various research methods to study DRSC comprehensively from the aspects of consumer preference, recycling mode, channel power, and consumer satisfaction, their research on recycling service level is still at the level of theoretical analysis, and has not made a more in-depth study of its specific decision issues.

### 2.2. Service Level Decisions

Improving service level has always been considered an important way to improve customer satisfaction and enterprise competitiveness. As early as 2009, some scholars conducted a more in-depth study on the definition of service in enterprise decisions. Wang (2009) thought that the term service is similar to “marketing efforts” in economics, which refers to the brand building activities of retailers, including hiring knowledgeable sales personnel, investing in advertisements, and improving the quality of goods and displays in stores [21]. Yan (2009) defined it as all forms of services that can enhance demand, including timely customer support, pre-sale suggestions, after-sales service, in-store advertising and promotion, technical assistance, return service, channel assembly service, and overall quality of shopping experience [22]. In addition, scholars have also conducted a more in-depth study on the influence of service level on enterprise decisions in supply chain from the aspects of channel differences, conflicts, and demands. Tsay (2004) used a formula to describe the influence of service level on demand and cost in the study of channel conflicts that may be caused by manufacturers introducing online channels. His description was widely quoted by scholars in this field in the following ten years. In recent years, the research on this problem is more concentrated [23]. Giri (2016) studied a supply chain model including one manufacturer and two retailers, considering the pricing, service, and coordination strategies of enterprises in the supply chain when the two retailers had price and service competition at the same time, and finally he proposed a price discount contract to achieve a win-win situation for enterprises [24]. Wang (2017) studied the pricing and service level decisions of enterprises under different dominance based on a supply chain model including two manufacturers and one retailer, and one of the manufacturers built an online channel. She pointed out that when retailers infinitely increase their service level, their demand decreases [25]. Zhang (2017) studied the return and refund of services in a dual-channel supply chain, and pointed out that when the power of manufacturer and retailer is equal, the retailer’s profit in the dual-channel is always higher than that in a single-channel [26]. Xie (2018) studied the recycling service of a dual-channel closed-loop supply chain including a manufacturer and a retailer, coordinated the supply chain by constructing a profit sharing contract, and pointed out that increasing the retailer’s profit sharing ratio would help to encourage retailers to improve their service level and profit [27]. Wang (2018) studied the supply chain model with only online recycling channels, and pointed out that the supply chain led by manufacturers would significantly improve the level of online recycling service and product prices by introducing fairness concerns [28].

In the above literature, deep research has been carried out on DRSC and service level decisions. However, they have not explored the pricing and service level decisions of DRSC in multi-regional situations. In fact, how to make differentiated service level decisions for different regions in DRSC has become an important practical problem that enterprise managers have to face, which is exactly what has not been involved in the previous literature. Different from previous studies, this paper studies the influence of consumer preference on the pricing and service level decisions of recycling enterprises with different pricing strategies in DRSC in multi-regional situations. The research conclusion can effectively improve the profits of enterprises and the supply chain system by providing management suggestions for enterprises.

## 3. Description of Notations

This paper studies the influence of consumer preference on pricing and service level decisions of recycling enterprises in DRSC mode in a multi-regional situation. Consumers in different cities A and B can choose to recycle their WEEE through traditional offline channels or online channels. In traditional offline channels, consumers first sell WEEE to TPR at the recycling price of *p_i_*, and then TPR sells WEEE to the recycling center at the offline transfer price of *w_i_*. In online channels, the recycling center outsources the whole online channel to TPP. Consumers need to sell WEEE to TPP at the price of *p_e_*, and then TPP sells WEEE to the recycling center at the online transfer price *w_e_*. In this process, TPP provides consumers with the recycling service level of *s_i_*. In addition, as mentioned in the first section, combined with the real situation, offline recycling price and online recycling service are affected by regional differences and there are differences. This means that when the regional differences in one region change, the decision and profits of recycling enterprises in another region may be affected. The recycling amount of consumers of online and offline channels depends on their preference for online channels, the recycling price of different channels, and the influence of service level [2,24]. Specifically, we assume that the basic number of WEEE held by consumers in a city is *α_i_*. At the same time, the online (offline) recycling amount is also affected by the positive correlation of the online (offline) recycling price and service level, and by the negative correlation of the recycling price and service level of its competitive channels. In addition, we also assume that the recycling center, TPR, and TPP follow Stackelberg game, and based on a large number of existing studies, we assume that the recycling center dominates, while TPR and TPP follow [1,5,10]. This means that in the game of the recycling center, TPR, and TPP, the recycling center makes a decision in its favor first; TPR and TPP can only make a decision after observing the decision of the recycling center. The major notations used in this paper are listed in Table 1, where *i* = 1 stands for city A and *i* = 2 stands for city B. 

In addition, similar to some existing studies by Wu [2], we assume that the relationship between service cost and service level satisfies *c_s_* = *ηs*^2^/2.This shows that the recycling service level has a significant positive correlation with service cost. In addition, we also assume that WEEE’s recycling is linearly related to online and offline recycling price and service level. Specifically, the recycling amount of different cities can be expressed as: *d_ri_* = (1 − *θ_i_*)*α_i_* + *m_i_p_i_* − *k_i_p_e_* − *n_i_s_i_*, *d_ei_* = *θ_i_α_i_* + *m_i_p_e_* − *k_i_p_i_* + *h_i_s_i_*.

## 4. Model Analysis

**Figure 1:**
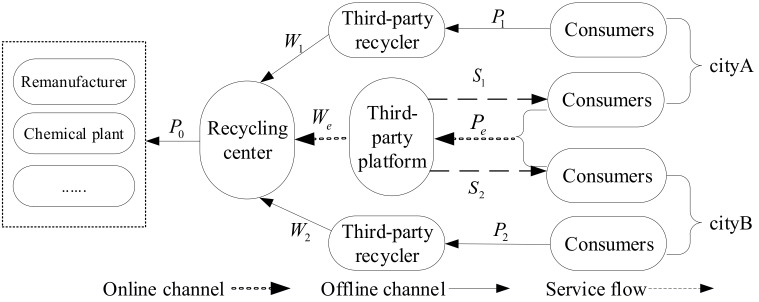
Depiction of the dual-channel reverse supply chain in multi-regional situations.

This section studies the DRSC mode which consists of one recycling center, one TPP, and two TPRs in different regions, including both online and offline recycling channels. As shown in Figure 1, in offline channels of this mode, TPRs in different regions sell WEEE recovered from consumers to the recycling center at a certain transfer price. In online recycling channels, TPPs recycle WEEE from different regions at the same online recycling price and sell WEEE to the recycling center at the online transfer price. Although the TPP sets the same online recycling price when facing multi-regional consumers, it sets different recycling service levels considering regional differences, such as the difference in the number of offline experience stores and the difference in the number and responsiveness of couriers who can provide door-to-door pick-up services. The main business of the recycling center is to decompose and dismantle WEEE, not to build the recycling platform or to provide a recycling service for consumers in the field of marketing. Therefore, the supply chain model can help the recycling center and TPP focus on their own business and give full play to their own advantages. In this section, three decision schemes are proposed for the recycling center that introduces online recycling channels: keeping offline transfer price unchanged, unifying all transfer prices, and maximizing its own profit. Numerical examples are used to verify the change trend of the profit of recycling enterprises under the three strategies when consumer preference changes. Finally, management suggestions are provided for the decision choice of recycling enterprises.

As the basis of later research, we first study the supply chain model without introducing online recycling channels in a multi-regional situation. By constructing the profit model of the recycling center and multi-regional TPR and applying Stackelberg game theory to solve it, the optimal pricing decision of each recycling enterprise can be obtained. Then, the profit functions of the recovery center and the two TPRs are П_1_ = *d_r_*_1_(*w*_1_ − *p*_1_) = (*α*_1_ + *m*_1_*p*_1_)(*w*_1_ − *p*_1_), П_2_ = *d_r_*_2_(*w*_2_ − *p*_2_) = (*α*_2_ + *m*_2_*p*_2_)(*w*_2_ − *p*_2_), П*_m_* = *d_r_*_1_(*p*_0_ − *w*_1_) + *d_r_*_2_(*p*_0_ − *w*_2_)= (*α*_1_ *+ m*_1_*p*_1_) (*p*_0_ − *w*_1_) + (*α*_2_ + *m*_2_*p*_2_) (*p*_0_ − *w*_2_).

According to the hypothesis, the recycling amount in region A is *d_r_*_1_ = *α*_1_ + *m*_1_*p*_1_, and that in region B is *d_r_*_2_ *= α*_2_ + *m*_2_*p*_2_. In Stackelberg game between the recycling center and TPR, the recycling center always occupies a dominant position, so the recycling center makes decisions first, and TPR makes decisions after observing the decisions of the recycling center. According to the backward induction method, the optimal recovery prices of the two TPRs are first solved.

**Property 1.**  *The objective function ∏_1_ (p_1_) is always concave with p_1_. The objective function ∏_2_ (p_2_) is always concave with p_2_.*

Property 1 (proofs can be found in Appendix A) indicates that for the functions ∏_1_ and ∏_2_, there is optimal *p*_1_ and *p*_2_ respectively, which makes ∏_1_ and ∏_2_ reach the maximum. Therefore, by solving the first partial derivatives of ∏_1_ for *p*_1_ and ∏_2_ for *p*_2_ respectively, and making them be 0, the optimal recovery price *p*_1_* of ∏_1_ can be obtained as follows:

$$p_{1}{}^{*}=\left(w_{1}m_{1}-\alpha_{1}\right)/2m_{1}$$

The optimal recovery price *p*_2_* of ∏_2_ is:

$$p_{2}{}^{*}=\left(w_{2}m_{2}-\alpha_{2}\right)/2m_{2}$$

Substituting the optimal offline recovery prices *p*_1_* and *p*_2_* of the two regions into the profit function of the recovery center, we can get:

$$\prod_{m}=\left(a_{1}+m_{1}p_{1}{}^{*}\right)(p_{0}-w_{1})+\left(a_{2}+m_{2}p_{2}{}^{*}\right)(p_{0}-w_{2})$$

**Property 2.**  *The objective function ∏_m_ (w_1_, w_2_) is always concave with w_1_ and w_2_.*

Property 2 (proofs can be found in Appendix A) indicates that for the function ∏_m_, there is optimal *w*_1_ and *w*_2_ respectively, which makes ∏_m_ reach the maximum. Therefore, by solving the first partial derivatives of ∏_m_ for *w*_1_ and *w*_2_ respectively, making them be 0, and combining them, the optimal offline transfer prices of ∏_m_ can be obtained as follows:

(1) $$w_{1}{}^{*}=\left(p_{0}m_{1}-\alpha_{1}\right)/2m_{1}$$

(2) $$w_{2}{}^{*}=\left(p_{0}m_{2}-\alpha_{2}\right)/2m_{2}$$

From the above, we can get the optimal transfer price of the recycling center before the introduction of online channels. Based on the above results, we propose three pricing strategies: keeping offline transfer price unchanged, unifying all transfer prices, and maximizing its own profit for recycling centers with online channels. These three pricing strategies are based on the classic research of Cattani [29] in 2006, aiming to help enterprises achieve a balance between existing channel partners and their own economic profits when introducing new Internet-based distribution channels. Relevant realistic cases are common in management practice [30]. For example, Hewlett Packard (HP) kept the prices of printers in all channels consistent in order to avoid supply chain conflicts after introducing online channels; Adidas even set the price of its sneakers slightly higher than that of traditional channels when it first established its online sales website. To sum up, this part of the study aims to maintain the coordination and stability of the supply chain, and at the same time provide reasonable decision support to improve the economic profit of recycling enterprises.

### 4.1. The Offline Transfer Price Is Unchanged

For quite a long time in the past, the recycling industry only had the offline channel with the recycling center–TPR as the core. However, when the recycling center tries to introduce the online channel based on the recycling center–TPP, this channel mode is bound to share some customer resources of offline channels, and directly affects the economic profit of the TPR and may lead to disharmony among channels. Therefore, in order to reduce the possible conflict with the TPR after the introduction of online recycling channels, the recycling center can choose the strategy of this section, that is, to control the offline transfer price of the TPR in each region to maintain the level before the introduction of online channels, such as (1) and (2).

Under this strategy, the recycling center, TPR, and TPP still obey Stackelberg game theory, that is, the recycling center occupies the dominant position and makes decisions first. TPR and TPP occupy the following position and make decisions after observing the decision of the recycling center. According to the backward induction method, we first solve the optimal decision of TPPs and multi-regional TPRs. To simplify the model without affecting the analysis of the research results in this paper and without losing generality, based on Wu (2020)’s research, we assume the following parameters: *m_i_* = *m_j_* = 2, *k_i_* = *k_j_* = 1, *h_i_* = *h_j_* = 2, *n_i_* = *n_j_* = 1. The profit function of the multi-regional TPR can be obtained as follows:

$$\Pi_{1}=\left(w_{1}{}^{*}-p_{1}\right)d_{r1}=\left[\left(2p_{0}-\alpha_{1}\right)/4-p_{1}\right]\left[\left(1-\theta_{1}\right)a_{1}+2p_{1}-p_{e}-s_{1}\right]\;\Pi_{2}=\left(w_{2}{}^{*}-p_{2}\right)d_{r2}=\left[\left(2p_{0}-\alpha_{2}\right)/4-p_{2}\right]\left[\left(1-\theta_{2}\right)a_{2}+2p_{2}-p_{e}-s_{2}\right]$$

The profit function of TPP is:

$$\begin{array}{l} \Pi_{e}=\left(w_{e}-p_{e}\right)\left(d_{e1}+d_{e2}\right)-c_{s1}-c_{s2}\\ =\left(w_{e}-p_{e}\right)\left[\theta_{1}\alpha_{1}+2p_{e}-p_{1}^{}+2s_{1}+\theta_{2}\alpha_{2}+2p_{e}-p_{2}^{}+2s_{2}\right]-\eta_{1}s_{1}^{2}/2-\eta_{2}s_{2}^{2}/2 \end{array}$$

**Property 3.**  *The objective function ∏_1_ (p_1_) is always concave with p_1_. The objective function ∏_2_ (p_2_) is always concave with p_2_. When η_1_ + η_2_ − 2η_1_η_2_ < 0, the objective function ∏_e_ (p_e_, s_1_, s_2_) is always concave with p_e_, s_1_, s_2_.*

Property 3 (proofs can be found in Appendix A) indicates that for functions ∏_1_ and ∏_2_, there is optimal *p*_1_ and *p*_2_ respectively, which makes ∏_1_ and ∏_2_ reach the maximum. For the function ∏_e_, there is optimal *p*_e_, *s*_1_, *s*_2_, which makes ∏_e_ reach the maximum. Therefore, by solving ∏_1_ for *p*_1_ and making it be 0, the optimal recovery price *p*_1_* of ∏_1_ can be obtained as follows:

(3) $$p_{1}{}^{*}=\frac{1}{8}\left(2p_{0}+2p_{e}+2s_{1}-3a_{1}+2a_{1}\theta_{1}\right)$$

Similarly, the optimal recovery price *p*_2_* of ∏_2_ can be obtained as follows:

(4) $$p_{2}{}^{*}=\frac{1}{8}\left(2p_{0}+2p_{e}+2s_{2}-3a_{2}+2a_{2}\theta_{2}\right)$$

By solving the first partial derivatives of ∏_e_ for *p*_e_, *s*_1_, *s*_2_ respectively, making them be 0, and then ombining them, the optimal decisions *p*_e_*, *s*_1_*, *s*_2_* of ∏_e_ can be obtained as follows:

(5) $$p_{e}{}^{*}=\left[4w_{e}\left(\eta_{1}\eta_{2}-\eta_{1}-\eta_{2}\right)+\eta_{1}\eta_{2}\left(p_{1}+p_{2}\right)\right]/2\left[-2\eta_{2}+\eta_{1}\left(4\eta_{2}-2\right)\right]$$

(6) $$s_{1}{}^{*}=\eta_{2}\left(-p_{1}-p_{2}+4w_{e}+a_{1}\theta_{1}+a_{2}\theta_{2}\right)/\left[-2\eta_{2}+\eta_{1}\left(4\eta_{2}-2\right)\right]$$

(7) $$s_{2}{}^{*}=\eta_{1}\left(-p_{1}-p_{2}+4w_{e}+a_{1}\theta_{1}+a_{2}\theta_{2}\right)/\left[-2\eta_{2}+\eta_{1}\left(4\eta_{2}-2\right)\right]$$

Construct the profit function of the recycling center and substitute *p*_1_* *p*_2_* *p*_e_*, *s*_1_*, *s*_2_* into the function, and we can get:

$$\begin{array}{l} \Pi_{m}=\left(p_{0}-w_{e}\right)\left(d_{e1}+d_{e2}\right)+\left(p_{0}-w_{1}\right)d_{r1}+\left(p_{0}-w_{2}\right)d_{r2}\\ =\left(p_{0}-w_{e}\right)\left(\theta_{1}\alpha_{1}+2p_{e}{}^{*}-p_{1}^{*}+2s_{1}{}^{*}+\theta_{2}\alpha_{2}+2p_{e}{}^{*}-p_{2}^{*}+2s_{2}{}^{*}\right)\\ +\left[p_{0}-\left(2p_{0}-\alpha_{1}\right)/4\right]\left[\left(1-\theta_{1}\right)\alpha_{1}+2p_{1}^{*}-p_{e}{}^{*}-s_{1}{}^{*}\right]+\left[p_{0}-\left(2p_{0}-\alpha_{2}\right)/4\right]\left[\left(1-\theta_{2}\right)\alpha_{2}+2p_{2}^{*}-p_{e}{}^{*}-s_{2}{}^{*}\right] \end{array}$$

**Property 4.**  *When 7η_1_ + 7η_2_ − 15η_1_η_2_ < 0, the objective function ∏_m_ (w_e_) is always concave with w_e_.*

Property 4 (proofs can be found in Appendix A) indicates that for the function ∏_m_, there is an optimal *w*_e_, which makes ∏_m_ reach the maximum. Therefore, by solving ∏_m_ for we and making it be 0, the optimal recovery price *w*_e_* of ∏_m_ can be obtained as follows:

(8) $$w_{e}{}^{*}=\left(7a_{1}\eta_{1}+7a_{2}\eta_{2}-7a_{2}\eta_{1}-7a_{1}\eta_{2}+224p_{0}\eta_{1}\eta_{2}-32a_{1}\eta_{1}\eta_{2}-32a_{2}\eta_{1}\eta_{2}-48a_{1}\eta_{1}\eta_{2}\theta_{1}-48a_{2}\eta_{1}\eta_{2}\theta_{2}\right)/448\eta_{1}\eta_{2}$$

Substituting the solutions of *p*_1_*, *p*_2_*, p_e_*, w_e_*, *s*_1_* and *s*_2_* into the profit functions of the recycling centers, TPPs, and TPRs of different regions, we can get the maximum profit of each recycling enterprise under the optimal pricing and service level decision under the 4.1 strategy.

**Corollary 1.**  *Under the 4.1 strategy, the optimal transfer price in online recycling channels is affected by the negative correlation of consumer preference for online recycling channels.*

Corollary 1 (proofs can be found in Appendix B) indicates that under the 4.1 strategy, when consumer preference for online channels increases in a certain region, or the recycling center expands the online recycling mode to regions with higher consumer preference, it can reduce the online transfer price to maximize its own profit.

### 4.2. Unify All Transfer Prices

Similar to the research in Section 4.1, in order to reduce conflicts among channels after the introduction of online channels and promote the coordination of the supply chain, in this section, we study the decision of the recycling center to unify all transfer prices. Under this strategy, the recycling center keeps all online transfer prices and multi-regional offline transfer prices equal, that is, *w* = *w*_1_ = *w*_2_ = *w*_e_. This can make TPP or multi-regional TPR avoid potential conflicts because other recycling enterprises get higher transfer price. The profit function of multi-regional TPR can be expressed as:

$$\Pi_{1}=\left(w-p_{1}\right)d_{r1}=\left(w-p_{1}\right)\left[\left(1-\theta_{1}\right)a_{1}+2p_{1}-p_{e}-s_{1}\right]\;\Pi_{2}=\left(w-p_{2}\right)d_{r2}=\left(w-p_{2}\right)\left[\left(1-\theta_{2}\right)a_{2}+2p_{2}-p_{e}-s_{2}\right]$$

Similar to the research in Section 4.1, since it has been proven that each recycling enterprise has its own optimal solution of decision that makes the profit reach the maximum, we directly solve the optimal solution of pricing and service level decisions of each enterprise. By solving the first partial derivative ∂∏_1_ /∂*p*_1_ of ∏_1_ for *p*_1_, making it be 0, and solving it in reverse, the optimal recycling price *p*_1_* of TPR in region A can be obtained:

(9) $$p_{1}{}^{*}=\frac{1}{4}\left(2w+p_{e}+s_{1}-a_{1}+a_{1}\theta_{1}\right)$$

Similarly, the optimal recycling price *p*_2_* of the TPR in region B is:

(10) $$p_{2}{}^{*}=\frac{1}{4}\left(2w+p_{e}+s_{2}-a_{2}+a_{2}\theta_{2}\right)$$

The profit function of the TPP is:

$$\begin{array}{l} \Pi_{e}=\left(w-p_{e}\right)\left(d_{e1}+d_{e2}\right)-c_{s1}-c_{s2}\\ =\left(w-p_{e}\right)\left[\theta_{1}\alpha_{1}+2p_{e}-p_{1}^{}+2s_{1}+\theta_{2}\alpha_{2}+2p_{e}-p_{2}^{}+2s_{2}\right]-\eta_{1}s_{1}^{2}/2-\eta_{2}s_{2}^{2}/2 \end{array}$$

By solving the first partial derivatives of ∏*_e_* for *p_e_*, *s*_1_ and *s*_2_ respectively, making them be 0, solving them in reverse, and combining them, the optimal pricing and service level decisions p_e_*, *s*_1_* and *s*_2_* of the TPP can be obtained as follows:

(11) $$p_{e}{}^{*}=\frac{-4w\eta_{2}+-4w\eta_{1}+\eta_{1}\eta_{2}\left(4w+p_{1}+p_{2}-a_{1}\theta_{1}-a_{2}\theta_{2}\right)}{-4\eta_{1}-4\eta_{2}+8\eta_{1}\eta_{2}}$$

(12) $$s_{1}{}^{*}=\frac{\eta_{1}\left(4w-p_{1}-p_{2}+a_{1}\theta_{1}+a_{2}\theta_{2}\right)}{-2\eta_{1}-2\eta_{2}+4\eta_{1}\eta_{2}}$$

(13) $$s_{2}{}^{*}=\frac{\eta_{2}\left(4w-p_{1}-p_{2}+a_{1}\theta_{1}+a_{2}\theta_{2}\right)}{-2\eta_{1}-2\eta_{2}+4\eta_{1}\eta_{2}}$$

Substituting the optimal solutions of ∏_1_, ∏_2_ and ∏*_e_* into ∏*_m_*, we can get:

$$\Pi_{m}=\left(p_{0}-w\right)\left(\alpha_{1}+p_{e}{}^{*}+p_{1}^{}{}^{*}+s_{1}{}^{*}+\alpha_{2}+p_{e}{}^{*}+p_{2}^{}{}^{*}+s_{2}{}^{*}\right)$$

By solving the first partial derivative ∂∏*_m_*/∂*w* of ∏*_m_* for *w*, making it be 0, and solving it in reverse, the optimal online transfer price w* of the recycling center can be obtained as follows:

(14) $$w^{*}=\frac{p_{0}\left(6\eta_{1}+6\eta_{2}-20\eta_{1}\eta_{2}\right)+a_{1}\left(-2\eta_{2}-2\eta_{1}+\eta_{2}\theta_{1}+\eta_{1}\theta_{1}+5\eta_{1}\eta_{2}\right)+a_{2}\left(-2\eta_{2}-2\eta_{1}+\eta_{2}\theta_{2}+\eta_{1}\theta_{2}+5\eta_{1}\eta_{2}\right)}{4\left(3\eta_{1}+3\eta_{2}-10\eta_{1}\eta_{2}\right)}$$

Substituting the solutions of p_i_*, p_j_*, p_e_*, w_e_*, s_i_*, and s_j_* into the profit functions of the recycling center, TPP, and TPR in different regions, the maximum profit of the enterprise under the optimal pricing and service level decision can be obtained.

### 4.3. Maximize Its Own Profits

Under this strategy, the recycling center occupies an absolute dominant position compared with the TPR and the TPP. This makes it possible to make strategies with the goal of maximizing its own profit, without considering the possible channel conflicts caused by the introduction of online channels. In Stackelberg game model, the recycling center makes a decision on its optimal online transfer price and multi-regional offline transfer price. After that, the followers, the TPP makes decisions on its online recycling price and multi-regional service level, and the multi-regional TPR makes decisions on its offline recycling price respectively. According to the reverse solution method, we first construct the profit model of followers TPR and TPP as follows:

$$\Pi_{1}=\left(w_{1}-p_{1}\right)d_{r1}=\left(w_{1}-p_{1}\right)\left[\left(1-\theta_{1}\right)a_{1}+2p_{1}-p_{e}-s_{1}\right]\;\Pi_{2}=\left(w_{2}-p_{2}\right)d_{r2}=\left(w_{2}-p_{2}\right)\left[\left(1-\theta_{2}\right)a_{2}+2p_{2}-p_{e}-s_{2}\right]\;\begin{array}{l} \Pi_{e}=\left(w_{e}-p_{e}\right)\left(d_{e1}+d_{e2}\right)-c_{1}-c_{2}\\ =\left(w_{e}-p_{e}\right)\left[\theta_{1}\alpha_{1}+2p_{e}-p_{1}^{*}+2s_{1}+\theta_{2}\alpha_{2}+2p_{e}-p_{2}^{*}+2s_{2}\right]-\eta_{1}s_{1}^{2}/2-\eta_{2}s_{2}^{2}/2 \end{array}$$

By solving the first partial derivative ∂∏_1_/∂*p*_1_ of ∏_1_ for *p*_1_, making it be 0, and solving it in reverse, the optimal recycling price *p*_1_* of the TPR in region A can be obtained as follows:

(15) $$p_{1}{}^{*}=\frac{1}{4}\left(2w_{1}+p_{e}+s_{1}-a_{1}+a_{1}\theta_{1}\right)$$

Similarly, the optimal recycling price *p*_2_* of the TPR in region B is:

(16) $$p_{2}{}^{*}=\frac{1}{4}\left(2w_{2}+p_{e}+s_{2}-a_{2}+a_{2}\theta_{2}\right)$$

By solving the first partial derivatives of ∏*_e_* for *p_e_*, *s*_1_ and *s*_2_ respectively, making it be 0, solving it in reverse, and combining them, the optimal pricing and service level decision p_e_*, *s*_1_* and *s*_2_* of the TPP can be obtained as follows:

(17) $$p_{e}{}^{*}=\frac{-4w_{e}\eta_{2}+-4w_{e}\eta_{1}+\eta_{1}\eta_{2}\left(4w_{e}+p_{1}+p_{2}-a_{1}\theta_{1}-a_{2}\theta_{2}\right)}{-4\eta_{1}-4\eta_{2}+8\eta_{1}\eta_{2}}$$

(18) $$s_{1}{}^{*}=\frac{\eta_{1}\left(4w_{e}-p_{1}-p_{2}+a_{1}\theta_{1}+a_{2}\theta_{2}\right)}{-2\eta_{1}-2\eta_{2}+4\eta_{1}\eta_{2}}$$

(19) $$s_{2}{}^{*}=\frac{\eta_{2}\left(4w_{e}-p_{1}-p_{2}+a_{1}\theta_{1}+a_{2}\theta_{2}\right)}{-2\eta_{1}-2\eta_{2}+4\eta_{1}\eta_{2}}$$

According to the reverse solution method, we construct the profit function of the recycling center. In this model, the profit of the recycling center includes the profit of offline recycling channels of two cities and the profit of online recycling channels outsourced to the TPP:

$$\begin{array}{l} \Pi_{m}=\left(p_{0}-w_{e}\right)\left(d_{e1}+d_{e2}\right)+\left(p_{0}-w_{1}\right)d_{r1}+\left(p_{0}-w_{2}\right)d_{r2}\\ =\left(p_{0}-w_{e}\right)\left[\theta_{1}\alpha_{1}+2p_{e}{}^{*}-p_{1}^{*}+2s_{1}{}^{*}+\theta_{2}\alpha_{2}+2p_{e}{}^{*}-p_{2}^{*}+2s_{2}{}^{*}\right]\\ +\left(p_{0}-w_{1}\right)\left[\left(1-\theta_{1}\right)\alpha_{1}+2p_{1}^{*}-p_{e}{}^{*}-s_{1}{}^{*}\right]+\left(p_{0}-w_{2}\right)\left[\left(1-\theta_{2}\right)\alpha_{2}+2p_{2}^{*}-p_{e}{}^{*}-s_{2}{}^{*}\right] \end{array}$$

By solving the first partial derivatives of ∏*_m_* for *w_e_*, *w*_1_ and *w*_2_ respectively, making it be 0, solving it in reverse, and combining them, the optimal pricing decisions of the recycling center w_e_*, *w*_1_* and *w*_2_* can be obtained as follows:

(20) $$w_{1}{}^{*}=\left[12p_{0}+\alpha_{1}\left(5\theta_{1}-7\right)-\alpha_{2}\left(\theta_{2}+1\right)\right]/24$$

(21) $$w_{2}{}^{*}=\left[12p_{0}+\alpha_{2}\left(5\theta_{2}-7\right)-\alpha_{1}\left(\theta_{1}+1\right)\right]/24$$

(22) $$w_{e}{}^{*}=6p_{0}-\alpha_{1}\left(1+\theta_{1}\right)-\alpha_{2}\left(1+\theta_{2}\right)/12$$

Substituting the solutions of *p*_1_*, *p*_2_*, p_e_*, w_e_*, *w*_1_*, *w*_2_*, *s*_1_*, *s*_2_* into the profit functions of the recycling center, TPP, and TPR in different regions, the maximum profit of each recycling enterprise under the optimal pricing and service level decisions can be obtained.

**Corollary 2.**  *Under the 4.3 strategy, for the recycling center, its optimal offline transfer price in a certain region is affected by the positive correlation of consumer preference for online recycling channels in the region, and affected by the negative correlation of consumer preference for online recycling channels in other regions. The optimal online transfer price of the recycling center is affected by the negative correlation of consumer preference for online recycling channels in different regions.*

Corollary 2 (proofs can be found in Appendix B) indicates that under the 4.3 strategy, when consumer preference for online channels in a certain region increases, or consumer preference for online channels in other regions decreases, or the recycling center expands online recycling channels to regions with lower consumer preference, it can maximize its profit by increasing the offline transfer price in this region. It can be seen that the change of consumer preference for online recycling channels in a certain region will affect the offline transfer price decision of the recycling center not only in this region, but also in other regions, and this kind of influence shows an opposite trend. In addition, the recycling center can also increase its profit by lowering the online transfer price.

**Corollary 3.**  When all other regional parameters except θ are equal: when 5θ_j_ − 37θ_i_ + 16 = 0, η = 2, or θ_i_ = 0.5, θ_j_ = 0.5, the optimal p_1_* under three different models remains equal. When 5θ_i_ − 37θ_j_ + 16 = 0, η = 2, or θ_i_ = θ_j_ = 0.5, the optimal p_2_* under three different models remains equal. When θ_i_ + θ_j_ = 1, the optimal p_e_^*^, s_1_^*^ and s_2_^*^ under three different models remain equal. When θ_i_ = θ_j_ = 0.5, the optimal w_1_* and w_2_^*^ under three different models remain equal. When θ_i_ = θ_j_ = 0.5, the profits of the recycling enterprises under three different pricing models remain equal.

Corollary 3 (proofs can be found in Appendix B) indicates that when other regional parameters are equal and consumer preferences for online recycling channels in different regions meet certain conditions, the decision and profits of enterprises under the three pricing models are equal. Because all recycling enterprises can get the same result no matter which pricing model they choose, the three pricing models are unified at this time.

In the above subsection, we constructed three kinds of pricing strategies in Section 4.1, Section 4.2, and Section 4.3, and solved and analyze the optimal decision and profit of enterprises under each strategy. In the next section, we further compare and analyze the model results under the three strategies through numerical analysis. By comprehensively comparing the pricing, service level decisions, and corporate profits of the enterprises under the three strategies, we can get some important management implications related to DRSC.

## 5. Numerical Example

### 5.1. θ Analysis

**Figure 2:**
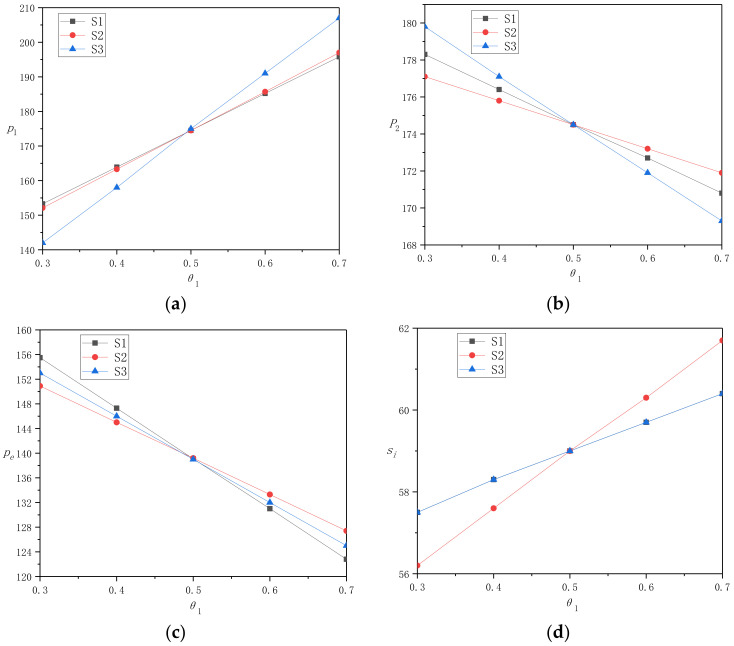
The change of decisions under three strategies as *θ*_1_ increases: (**a**) *p*_1_, (**b**) *p*_2_, (**c**) *p*_e_, (**d**) *s_i_*.

**Figure 3:**
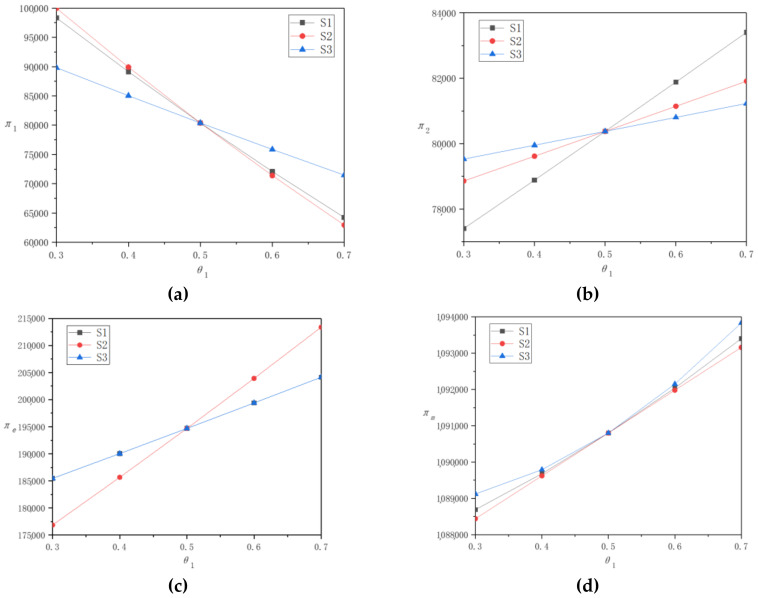
The change of profits under three strategies as *θ*_1_ increases: (**a**) ∏_1_, (**b**) ∏_2_, (**c**) ∏*_e_*, (**d**) ∏*_m_*.

In this section, we use the example analysis to simulate the research results in Section 4, in order to verify the existing research results, find new laws, and provide management enlightenment for enterprises. Specifically, we assume that *a*_1_ = *a*_2_ = 500, *p*_0_ = 1000, *θ*_2_ = 0.5, *η*_1_ = *η*_2_ = 8, and take *θ*_1_ to increase gradually from 0.3 to 0.7 at a rate of 0.1. By inputting the above data into Mathematica software, the research results verify that the promotion of consumer preference for online recycling channels in region A will have an impact on the decision and profit of the recycling center, TPP, and multi-regional TPR in a multi-regional situation. According to the simulation results, we draw Table 2, Table 3 and Table 4 to analyze the effect of consumer preference in region A on the decision and profit of recycling enterprises under the three strategies of keeping transfer price unchanged, unifying all transfer prices, and maximizing profit. Secondly, according to Figure 2 and Figure 3, we compare the change rules of decision and profit of recycling enterprises under three pricing strategies. To make the statement clearer, we use S1, S2, and S3 to represent the strategies in Section 4.1, Section 4.2, and Section 4.3, respectively.

From Table 2, it can be seen that with the increase of consumer preference for online recycling channels in region A, under the S1 strategy, the recycling center needs to reduce the online transfer price to increase its own profit. TPP needs to reduce the online recycling price and improve the online recycling service level in all regions, so as to achieve a significant increase in profits. Secondly, for the TPR in region A, the optimal offline recycling price increases with the increase of *θ*_1_, while its profit decreases monotonically. In addition, for the TPR in region B, the optimal offline recycling price decreases slightly with the increase of *θ*_1_, while its profit increases monotonically. It can be seen from the above that under this strategy, the change of consumer preference for online recycling channels in region A will not only affect the changes of profit and decision of the TPR, TPP, and recycling center in this region, but also affect the changes of profit and decision of the TPR in region B. Finally, it can be seen from the above table that under this strategy, the total profit of the supply chain system will decrease slightly with the increase of consumer preference for online recycling channels in region A. 

From Table 3, it can be seen that with the increase of consumer preference for online recycling channels in region A, under S2 strategy, the recycling center needs to slightly reduce online and offline transfer prices to improve its own profit. The TPP needs to reduce the online recycling price and improve the multi-regional online recycling service level so as to achieve a significant increase in profit. Secondly, for the TPR in region A, the optimal offline recycling price increases with the increase of *θ*_1_, while its profit decreases monotonically. In addition, for the TPR in region B, the optimal offline recycling price decreases slightly with the increase of *θ*_1_, while its profit increases monotonically. It can be seen from the above that under this strategy, the change of consumer preference for online recycling channels in region A will not only affect the changes of profit and decision of the TPR, TPP, and recycling center in this region, but also affect changes of the profit and decision of the TPR in region B. Finally, it can be seen from the above table that under this strategy, the total profit of the supply chain system will increase slightly with the increase of consumer preference for online recycling channels in region A. 

From Table 4, it can be seen that with the increase of consumer preference for online recycling channels in region A, under S3 strategy, the recycling center needs to slightly reduce offline and online transfer prices in region B, and increase offline transfer price in region A to improve its own profit. The TPP needs to reduce the online recycling price and improve multi-regional online recycling service level so as to achieve a significant increase in profit. Secondly, for the TPR in region A, the optimal offline recycling price increases with the increase of *θ*_1_, while its profit decreases monotonically. In addition, for the TPR in region B, the optimal offline recycling price decreases slightly with the increase of *θ*_1_, while its profit increases monotonically. It can be seen from the above that under this strategy, the change of consumer preference for online recycling channels in region A will not only affect the changes of profit and decision of the TPR, TPP, and recycling center in this region, but also affect the changes of the profit and decision of the TPR in region B. Finally, it can be seen from the above table that under this strategy, the total profit of the supply chain system will increase slightly with the increase of consumer preference for online recycling channels in region A.

After using tables to show and analyze the optimal decisions and profit of recycling enterprises under the three strategies, we vertically compare the decision of recycling enterprises under S1, S2, and S3 strategies in Figure 2, and compare their profit under each strategy in Figure 3. Intuitive observation shows that the numerical results in Figure 2 and Figure 3 verify Corollary 3. That is, if all other regional parameters except *θ* are equal, when *θ* satisfies certain conditions, the decision and profit of recycling enterprises under the three pricing models are equal. Next, we compare and analyze the change trend of decision and profit of each enterprise under the three pricing models before and after the turning point of *θ*.

Figure 2 shows the changing trend of the optimal decision of each recycling enterprise under the three pricing strategies in Section 4, with the increase of consumer preference for online channels in region A under DRSC mode. First of all, it can be seen from Figure 2a that when consumers in region A have higher preference for online recycling channels, but lower than those in region B, the offline recycling price of TPR in region A under S1 strategy will be higher than that under S3 strategy. On the contrary, the offline recycling price of TPR in region A under S3 strategy will be higher than that under S1 strategy, and the offline recycling price in region A under S2 strategy is always in the middle level.

It can be seen from Figure 2b that when consumers in region A have higher preference for online recycling channels but lower than those in region B, the offline recycling price of the TPR in region B under S3 strategy will be higher than that under S2 strategy. On the contrary, the offline recycling price in the TPR in region B under S2 strategy will be higher than that under S3 strategy, and the offline recycling price of the TPR in region B under S1 strategy is always in the middle level.

It can be seen from Figure 2c that when consumers in region A have a higher preference for online recycling channels but lower than those in region B, the online recycling price of the TPP in region B under S1 strategy will be higher than that under S2 strategy. When consumers in region A have higher preference for online recycling channels than those in region B, the online recycling price of the TPP under S2 strategy will be higher than the offline recycling price of the TPP under S1 strategy. The online recycling price under S3 strategy is always in the middle level.

It can be seen from Figure 2d that the change trend of the optimal service level of the TPP under S1 and S3 strategies is consistent all the time. When consumers in region A have a higher preference for online recycling channels, but lower than those in region B, the optimal service level of the TPP under S1 and S3 strategies will be higher than that under S2 strategy. On the contrary, the optimal service level of the TPP under S1 and S3 strategies will be lower than that under S2 strategy. 

It can be seen from Figure 3a that when consumers in region A have a higher preference for online recycling channels but lower than those in region B, the profit of the TPR in region A under S2 strategy will be higher than that under S3 strategy. On the contrary, the profit of the TPR in region A under S2 strategy will be lower than that under S3 strategy. Under S1 strategy, the profit of the TPR in region A is always in the middle level. 

It can be seen from Figure 3b that when consumers in region A have a higher preference for online recycling channels but lower than those in region B, the profit of the TPR in region B under S3 strategy will be higher than that under S1 strategy. On the contrary, the profit of the TPR of region B under S3 strategy will be lower than that under S1 strategy. Under S2 strategy, the profit of the TPR in region B is always in the middle level. 

It can be seen from Figure 3c that change trend of the profits of the TPP under S1 and S3 strategies is consistent all the time. When consumers in region A have a higher preference for online recycling channels but lower than those in region B, the profits of the TPP under S1 and S3 strategies are higher than those under S2 strategy. On the contrary, the profit of the TPP under S1 and S3 strategies will be lower than those under S2 strategy. 

It can be seen from Figure 3d that when consumers in region A have lower or higher preference for online recycling channels than those in region B, the profit of the recycling center under S3 strategy is always higher than that under S1 strategy, and the profit of the recycling center under S1 strategy is always higher than that under S2 strategy. When consumers in both region A and region B have equal preference for online recycling channels, the profit of the recycling center under the three strategies remain equal. 

### 5.2. Discussion

In this section, we further analyze and discuss the data in Section 5.1, in order to provide management suggestions for enterprise decision-makers in the case of changes in various practical factors. 

First, as the leader in the supply chain, the recycling center makes the decision first and it can always get the maximum profit when choosing S3 strategy from Figure 3d. At the same time, it can be seen from Table 4 that when consumer preference for online channels in a certain region increases, the optimal decision of the recycling center choosing S3 strategy is to increase local offline transfer price, and to reduce the online transfer price and the offline transfer price in other regions. At this time, its profit is monotonically increasing. This shows that the change of regional difference factors will have a significant impact on the decision and profit of the recycling center. However, when the TPR of offline channels is very resistant to the introduction of online channels by the recycling center, the recycling center needs to consider this reality when making decisions, so as to avoid TPR malicious bidding, fraud, and other behaviors, and further lead to supply chain conflicts. In this paper, two strategies, S1 and S2, are proposed for the recycling center in this case. Through comparative analysis, the profit of the recycling center under S1 strategy is always higher than that under S2 strategy. Under S1 strategy, when regional consumer preference for online channels increases, the optimal decision of the recycling center is to reduce the online transfer price, and its own profit is monotonically increasing.

Secondly, it can be seen from Figure 2a that for the TPR in different cities, when the consumer preference for online channels in city A increases, the optimal decision of the TPR in city A is to reduce the offline recycling price. As a follower of the game, it has no first-mover advantage, so its profit monotonically decreases, regardless of whether the recycling center chooses S1 or S3 strategy, which can also be seen in Figure 3a. In addition, when consumer preference in city A is lower than that in city B, the profit of the TPR in region A under S1 strategy will be higher than that under S3 strategy, otherwise, the profit of the TPR under S3 strategy will be higher. Examining the influence of consumer preference increase in city A on city B, the opposite conclusion can be drawn from Figure 2b and Figure 3b. This means that the change of difference factors in one region will not only affect the pricing decision and profit of the TPR in this region, but also have a significant impact on the TPR in another region. It also shows that the recycling center choosing S1 strategy will help to protect the TPR in cities with lower consumer preference, reduce their profit loss, and at the same time restrain the increase of TPR profit in regions with higher consumer preference. This is undoubtedly a strategy to effectively promote the coordination of the supply chain system. 

Thirdly, it can be seen from Figure 3c that the profits of the TPP under S1 and S3 strategies formulated by the recycling center are equal. Meanwhile, as can be seen from Figure 2c,d, with the increase of consumer preference in city A, the optimal online recycling price of the TPP decreases monotonically, while the optimal service level increases monotonically. The increase of online recycling price can further increase the profit of the TPP recycling unit WEEE. At the same time, although the TPP improves the service level and increases the recycling service cost, it also greatly increases the recycling amount of online channels. Taken together, the TPP’s decisions can greatly improve its own profits. In addition, according to the research of Section 4.1 and Section 4.3, combined with the numerical example study, it is easy to find if the service level of the TPP for multi-regional differentiation has nothing to do with consumer preference, but depends on the service cost coefficient of different regions. This shows that not all changes in regional differences have an impact on enterprise decision-making. In the example in this section, TPP always provides the same service level for multiple regions, assuming that the service cost coefficients of multiple regions are equal. 

## 6. Conclusions

In this paper, the recycling enterprises in dual channel reverse supply chain in multi-regional situation are taken as the research object, and the optimal pricing and service level decisions of recycling enterprises are considered when consumer preference for online channels changes in a certain region. Firstly, we construct the pricing decision model for each recycling enterprise with only offline channels in multi-regional situations. Then, after the introduction of the third-party recycling platform in the recycling center, three pricing strategies are designed to keep the offline transfer price unchanged, unify all transfer prices, and maximize their own profits. According to Stackelberg game theory, we construct and solve the decision model of each recycling enterprise. Finally, we use a numerical example to verify the influence of the change of consumer preference for online channels on the pricing decision, service level decision, and profit of each enterprise under the three pricing strategies. At the same time, by comparing the profits of each enterprise under the three strategies, this paper puts forward management decision suggestions for them from the perspective of economic benefit and promoting supply chain coordination. Several significant managerial insights were obtained as follows:

(1) At the beginning of the introduction of online channels in the recycling center, it may be protested, dissatisfied, or even maliciously defrauded by the TPR due to the segmentation of the offline channel market. In order to reduce the conflicts among channels and promote the coordination of supply chain, this paper puts forward two strategies for the recycling center: keeping the offline transfer price unchanged and unifying all transfer prices. Through comparison and verification, the strategy of keeping offline transfer price unchanged can always bring higher economic profit to the recycling center. When there is no risk of conflict in the supply chain system, the recycling center can make a pricing decision with the goal of maximizing its own profits. In addition, when consumer preference for online channels in a certain region increases, the optimal decision of the recycling center under S1 strategy is to reduce the online transfer price; the optimal decision under S3 strategy is to increase the offline transfer price in this region, and reduce the online transfer price and the offline transfer price in other regions.

(2) The TPR needs to adjust its pricing strategy according to the change of local consumer preference. When consumer preference for online channels increases, it should increase its offline recycling price to reduce the reduction in the recycling amount and profit of offline channels. At the same time, the TPR also needs to pay attention to the change of consumer preference in other regions because this will also affect its own decision and profit. When consumer preference for online channels increases in other regions, the TPR can moderately reduce the offline recycling price to further increase its own profits.

(3) Whether the recycling center chooses S1 or S3 strategy, it will not affect the profit of the TPP. The increase of consumer preference for online channels will significantly increase the profits of the TPP and the recycling center. At this time, the optimal strategy of the TPP is to reduce the online recycling price and improve the recycling service level. The reduction of online recycling price can reduce its own expenditure. Although the improvement of service level will increase service cost, it will also further increase online recycling amount. In addition, whether the TPP makes differentiated service level decisions for multiple regions is only related to the service cost coefficients of each region and is not affected by consumer preference. Finally, the TPP and the recycling center can take various measures such as advertising and policy guidance to actively promote consumer preference for online channels in various regions, so as to improve their own profits.

Aiming at the decision-making optimization of recycling enterprises in DRSC, this paper constructs, solves, and analyzes the promotion of three kinds of pricing models to the optimal pricing, service level decisions, and economic profit of enterprises by considering the characteristics of consumers’ channel preference factors and online recycling service level decision. In this paper, there are still some deficiencies in supply chain structure and objective function, which need to be further expanded by scholars in the future. On the one hand, the study object of this paper is the structure of reverse supply chain. However, the remanufacturing problem involving online recycling has been developed rapidly in China. Therefore, in the future, scholars can extend this research to the pricing problem of closed-loop supply chain considering the recovery conversion rate and remanufactured product sales. On the other hand, this study takes the economic benefits of enterprises as the objective function in the recycling process and has not yet involved the research objectives of considering environmental benefits such as carbon emissions and carbon footprints. This problem can also be used as a research direction in the future.

## Figures and Tables

**Table 1:** Notation and explanations. WEEE, waste electrical and electronic equipment; TPR, third-party recycler; TPP, third-party platform.

Notation	Explanation
*d_ri_*	Offline recycling amount (*i* = 1,2)
*d_ei_*	Online recycling amount (*i* = 1,2)
*θ_i_*	Consumer preference for online channels (*i*= 1,2)
*p*_0_	Unit income of the recycling center by remanufacturing or reselling WEEE
*p_i_*	Offline recycling price of TPR (*i* = 1,2)
*p_e_*	Online recycling price of the recycling center or TPP
*w_i_*	Offline transfer price of the recycling center (*i* = 1,2)
*w_e_*	Online transfer price of the recycling center
*s_i_*	Service level of online recycling channels, provided by the recycling center or TPP (*i* = 1,2)
*c_si_*	Service cost of online recycling channels (*i* = 1,2)
*η_i_*	Service cost coefficient (*η* > 0, *i* = 1,2)
*a_i_*	Basic value of the recycling market (*i* = 1,2)
*m_i_*	Elasticity coefficient (*m_i_* > 0, *i* = 1,2) of recycling amount affected by its own channel recycling price
*k_i_*	Elasticity coefficient (*m_i_* > *k_i_* > 0, *i* = 1,2) of recycling amount affected by the recycling price of competitive channels
*h_i_*	Elasticity coefficient (*h_i_* > 0, *i* = 1,2) of recycling amount affected by the service level of its own channels
*n_i_*	Elasticity coefficient (*h_i_* > *n_i_* > 0, *i* = 1,2) of recycling amount affected by the service level of competitive channels
∏*_m_*	profit of the recycling center
∏*_i_*	profit of TPR (*i* = 1,2)
∏*_e_*	profit of TPP

**Table 2:** The effect of *θ*_1_ on decisions by applying strategy S1.

*θ*_1_	*w_e_*	*p*_1_	*p*_2_	*p_e_*	*s*_1_	*s*_2_	*∏*_1_	*∏*_2_	*∏_e_*	*∏_m_*
0.3	385.7	153.3	178.3	155.5	57.5	57.5	98,330	77,407	185,454	1,088,690
0.4	380.4	163.9	176.4	147.3	58.3	58.3	89,128	78,885	190,042	1,089,680
0.5	375.0	174.5	174.5	139.1	59.0	59.0	80,378	80,378	194,687	1,090,800
0.6	369.6	185.2	172.7	131.0	59.7	59.7	72,080	81,884	199,387	1,092,040
0.7	364.3	195.8	170.8	122.8	60.4	60.4	64,234	83,405	204,144	1,093,400

**Table 3:** The effect of *θ*_1_ on decisions by applying strategy S2.

*θ*_1_	*w*	*p*_1_	*p*_2_	*p_e_*	*s*_1_	*s*_2_	*∏*_1_	*∏*_2_	*∏_e_*	*∏_m_*
0.3	375.7	152.1	177.1	150.9	56.2	56.2	99,963	78,857	176,846	1,088,440
0.4	375.3	163.3	175.8	145.0	57.6	57.6	89,904	79,616	185,659	1,089,620
0.5	375.0	174.5	174.5	139.2	59.0	59.0	80,378	80,378	194,687	1,090,800
0.6	374.7	185.7	173.2	133.3	60.3	60.3	71,385	81,144	203,928	1,091,980
0.7	374.3	197.0	171.9	127.4	61.7	61.7	62,926	81,913	213,384	1,093,160

**Table 4:** The effect of *θ*_1_ on decisions by applying strategy S3.

*θ*_1_	*w*_1_	*w*_2_	*w_e_*	*p*_1_	*p*_2_	*p_e_*	*s*_1_	*s*_2_	*∏*_1_	*∏*_2_	*∏_e_*	*∏_m_*
0.3	354	379	383	142.3	179.8	153.1	57.5	57.5	89,812	79,529	185,454	1,089,120
0.4	365	377	379	158.4	177.1	146.1	58.3	58.3	85,030	79,953	190,042	1,089,790
0.5	375	375	375	174.5	174.5	139.2	59.0	59.0	80,378	80,378	194,687	1,090,800
0.6	385	373	371	190.7	171.9	132.2	59.7	59.7	75,857	80,804	199,387	1,092,150
0.7	396	371	367	206.8	169.3	125.2	60.4	60.4	71,467	81,231	204,144	1,093,830

## Appendix A

**Proof of Property 1:**  First, by solving the first partial derivative of ∏*_i_* for *p_i_*, we can get:

$$\partial \prod_{1}/\partial p_{1}=-2m_{1}p_{1}+w_{1}m_{1}-\alpha_{1}$$

Secondly, by solving the second-order partial derivative of ∏*_I_* for *p_i_*, we can obtain:

$$\partial^{2}\prod_{1}/\partial p_{1}{}^{2}=-2m_{1}$$

Because *m*_1_ > 0, there is a constant ∂^2^∏_1_/∂*p*_1_^2^ < 0 Therefore, the profit function ∏_1_ of the third-party recycler is convex function for *p*_1_ In the same way, it can be proved that the function ∏_2_ is strictly convex for *p*_2_. This ends the proof. □

**Proof of Property 2:**  By solving the first and second partial derivatives of ∏*_m_* for *w*_1_, we can obtain:

∂∏m∂w1=p0m12−α1−(2w1m1−α12)∂2∏m∂w12=−m1≺0

By solving the first and second partial derivatives of ∏*_m_* for *w*_2_, we can obtain:

∂∏m∂w2=p0m22−α2−(2w2m2−α22)∂2∏m∂w22=−m2≺0

Therefore, the Hessian Matrix for constructing ∏*_m_* (*w*_1_, *w*_2_) is as follows:

H(∏m)=∂2∏m∂w12∂2∏m∂w1∂w2∂2∏m∂w2∂w1∂2∏m∂w22=−m100−m2

The first-order principal sub-formula of Hessian Matrix H(∏*_m_*) is −*m*_1_ < 0, and the second-order principal sub-formula is *m*_1_, *m*_2_ > 0. Therefore, the profit function ∏*_m_* of the recycling center is strictly joint convex function for *w*_1_ and *w*_2_. This ends the proof. □

**Proof of Property 3:**  Firstly, by solving the first partial derivative of ∏_1_ to *p*_1_, we can obtain:

∂∏1∂p1=pe−2p1+s1+2−p1+2p0−a14−a11−θ1

Secondly, by solving the second-order partial derivative of ∏_1_ for *p*_1_, we can obtain

∂2∏1∂p12=−4

Since −4 > 0, there exists a constant of ∂^2^∏_1_/∂*p*_1_^2^ < 0 Therefore, the profit function ∏_1_ of TPR is convex function for *p*_1_ In the same way, it can be proved that the function ∏_2_ is strictly convex for *p*_2_. The first-order and second-order partial derivatives of ∏*_e_* for *p_e_*, *s*_1_, *s*_2_ are solved respectively, and the Hessian Matrix of ∏*_e_* (*p_e_*, *s*_1_, *s*_2_) is drawn as follows:

H(∏e)=∂2∏e∂pe2∂2∏e∂pe∂s1∂2∏e∂pe∂s2∂2∏e∂s1∂pe∂2∏e∂s12∂2∏e∂s1∂s2∂2∏e∂s2∂pe∂2∏e∂s2∂s1∂2∏e∂s22=−8−2−2−2−ηi0−20−ηj

Since the first order principal sub-formula of Hessian Matrix H(∏*_m_*) is less than 0 and the second order principal sub-formula *η_i_η_j_* is greater than 0, when the third order principal sub-formula is 2*η*_1_ + 2*η*_2_ − 4*η*_1_*η*_2_ < 0, then the function ∏_m_ is joint convex function for *p_e_*, *s*_1_, *s*_2_. This ends the proof. □

**Proof of Property 4:**  By solving the first and second partial derivatives of ∏*_m_* for *w*_e_, we can obtain:

∂∏m∂we=−−224p0−2weη1η2+a17η2−7η1+16η1η22+3θ1+a27η1−7η2+16η1η22+3θ28−7η1−7η2+15η1η2∂2∏m∂we2=−448η1η28−7η1−7η2+15η1η2

Then, when 7*η_1_* + 7*η_2_* − 15*η_1_η_2_ <* 0, the second partial derivative of ∏*_m_* for *w*_e_ is always less than 0, the objective function ∏_m_ (w_e_) is always concave with w_e_. This ends the proof. □

## Appendix B

**Proof of Corollary 1:**  According to the research results in Section 4.1, the optimal online transfer price of the recycling center is:

$$w_{e}{}^{*}=\left(7a_{1}\eta_{1}+7a_{2}\eta_{2}-7a_{2}\eta_{1}-7a_{1}\eta_{2}+224p_{0}\eta_{1}\eta_{2}-32a_{1}\eta_{1}\eta_{2}-32a_{2}\eta_{1}\eta_{2}-48a_{1}\eta_{1}\eta_{2}\theta_{1}-48a_{2}\eta_{1}\eta_{2}\theta_{2}\right)/448\eta_{1}\eta_{2}$$

Let *w_e_** solve the first-order partial derivatives of *θ*_1_ and *θ*_2_ respectively, and it is easy to obtain that the coefficients are all less than 0. Therefore, the optimal online transfer price of the recycling center is affected by the negative correlation of consumer preference. This ends the proof. □

**Proof of Corollary 2:**  Let *w*_1_* solve the first-order partial derivatives of *θ*_1_ and *θ*_2_ respectively, and it is easy to obtain that the coefficient of 5*a*_1_/24 is always greater than 0 when the partial derivative of *θ*_1_ is solved and the coefficient of -*a*_2_/24 is always less than 0 when the partial derivative of *θ*_2_ is solved. A similar conclusion can be obtained by solving the partial derivatives of *θ*_1_ and *θ*_2_ for *w*_2_*. Therefore, the optimal offline transfer price of the recycling center is affected by the positive correlation of consumer preference for online recycling channels in this region and negative correlation of consumer preference for online recycling channels in other regions. Let *w_e_** solve the first-order partial derivatives of *θ*_1_ and *θ*_2_ respectively, and it is easy to obtain that the coefficients are always less than 0. Therefore, it can be seen that the optimal online transfer price of the recycling center is affected by the negative correlation of consumer preference in different regions. This ends the proof. □

**Proof of Corollary 3:**  Make all regional difference factors equal except *θ*, namely *a*_1_ = *a*_2_ = *a*,*η*_1_ = *η*_2_ = *η*, and substitute them into the decision function of each enterprise. Study the conditions of keeping the optimal *p*_1_* equal under three pricing models. Let (3) = (9) = (15), and we can get the condition that: *a* = 0; 5*θ_j_* − 37*θ*_i_ + 16 = 0, *η* = 2; *θ_i_* = 0.5, *θ_j_* = 0.5. According to the hypothesis that *a* > 0 is required, a = 0 is excluded. Therefore, it can be concluded that conditions for keeping the optimal *p*_1_^*^ equal under the three pricing models are 5*θ_j_* − 37*θ*_i_ + 16 = 0, *η* = 2; *θ_i_* = 0.5, *θ_j_* = 0.5. Study the conditions of keeping the optimal *p*_2_* equal under three pricing models. Let (4) = (10) = (16), and we can get the condition that: *a* = 0; 5*θ_i_* − 37*θ*_j_ + 16 = 0, *η* = 2; *θ_i_* = 0.5, *θ_j_* = 0.5. According to the hypothesis that *a* > 0 is required, *a* = 0 is excluded. Therefore, it can be concluded that conditions for keeping the optimal *p*_2_* equal under the three pricing models are 5*θi* − 37*θ*j + 16 = 0, *η* = 2; *θ_i_* = 0.5, *θ_j_* = 0.5. Study the conditions of keeping the optimal *p_e_** equal under three pricing models. Let (5) = (11) = (17), and we can get the condition that: *a* = 0; *θ_i_* + *θ_j_* = 1. According to the hypothesis that *a* > 0 is required, *a* = 0 is excluded. Therefore, it can be concluded that conditions for keeping the optimal *p_e_** equal under the three pricing models are *θ_i_* + *θ_j_* = 1. Study the conditions of keeping the optimal *s*_1_^*^ equal under three pricing models. Let (6) = (12) = (18), and we can get the condition that: *a* = 0; *θ_i_* + *θ_j_* = 1. According to the hypothesis that *a* > 0 is required, *a* = 0 is excluded. Therefore, it can be concluded that conditions for keeping the optimal *s*_1_* equal under the three different strategies are *θ_i_* + *θ_j_* = 1. In the same way, we can get that condition for keeping the optimal *s*_2_* equal under the three pricing models are *θ_i_* + *θ_j_* = 1. Study the conditions that keeping the optimal *w*_1_* equal under three pricing models. Let (1) = (14) = (20), and we can get the condition that: *a* = 0; *θ_i_* = *θ_j_* = 0.5. According to the hypothesis, that *a* > 0 is required, *a* = 0 is excluded. Therefore, it can be concluded that conditions for keeping the optimal *w*_1_* equal under the three different strategies are *θ_i_* = *θ_j_* = 0.5. In the same way, we can get that conditions for keeping the optimal *w*_2_* equal under the three pricing models are *θ_i_* = *θ_j_* = 0.5. All the above conditions are jointly established, and it can be seen that when *θ_i_* = *θ_j_* = 0.5, all decisions of enterprises under the three pricing models remain equal. All the decisions are equal, which directly leads to the same profits under the three pricing models. Therefore, the condition for each recycling enterprise to keep profits equal under the three pricing models is *θ_i_* = *θ_j_* = 0.5. This ends the proof. □
